# Comparative Epidemiologic Characteristics of Pertussis in 10 Central and Eastern European Countries, 2000-2013

**DOI:** 10.1371/journal.pone.0155949

**Published:** 2016-06-03

**Authors:** Ulrich Heininger, Philippe André, Roman Chlibek, Zuzana Kristufkova, Kuulo Kutsar, Atanas Mangarov, Zsófia Mészner, Aneta Nitsch-Osuch, Vladimir Petrović, Roman Prymula, Vytautas Usonis, Dace Zavadska

**Affiliations:** 1 University of Basel Children's Hospital (UKBB), Basel, Switzerland; 2 Service d'Hygiène, Epidémiologie et Prévention, Hôpital Edouard Herriot, Hospices Civils de Lyon, Lyon, France; 3 Department of Epidemiology, University of Defence, Hradec Kralove, Czech Republic; 4 Faculty of Public Health, Slovak Medical University, Bratislava, Slovakia; 5 Estonian Health Board, Tallinn, Estonia; 6 Hospital for Infectious Diseases, Sofia, Bulgaria; 7 National Institute of Health Promotion, Paediatric Directorate, Budapest, Hungary; 8 Department of Family Medicine, Medical University of Warsaw, Warsaw, Poland; 9 Institute for Public Health of Vojvodina, Faculty of Medicine, University of Novi Sad, Novi Sad, Serbia; 10 University Hospital, Hradec Kralove, Czech Republic; 11 Clinic of Children’s Diseases, Faculty of Medicine, Vilnius University, Vilnius, Lithuania; 12 Department of Paediatrics, Riga Stradins University, Riga, Latvia; Universidad Nacional de la Plata, ARGENTINA

## Abstract

We undertook an epidemiological survey of the annual incidence of pertussis reported from 2000 to 2013 in ten Central and Eastern European countries to ascertain whether increased pertussis reports in some countries share common underlying drivers or whether there are specific features in each country. The annual incidence of pertussis in the participating countries was obtained from relevant government institutions and/or national surveillance systems. We reviewed the changes in the pertussis incidence rates in each country to explore differences and/or similarities between countries in relation to pertussis surveillance; case definitions for detection and confirmation of pertussis; incidence and number of cases of pertussis by year, overall and by age group; population by year, overall and by age group; pertussis immunization schedule and coverage, and switch from whole-cell pertussis vaccines (wP) to acellular pertussis vaccines (aP). There was heterogeneity in the reported annual incidence rates and trends observed across countries. Reported pertussis incidence rates varied considerably, ranging from 0.01 to 96 per 100,000 population, with the highest rates generally reported in Estonia and the lowest in Hungary and Serbia. The greatest burden appears for the most part in infants (<1 year) in Bulgaria, Hungary, Latvia, Romania, and Serbia, but not in the other participating countries where the burden may have shifted to older children, though surveillance of adults may be inappropriate. There was no consistent pattern associated with the switch from wP to aP vaccines on reported pertussis incidence rates. The heterogeneity in reported data may be related to a number of factors including surveillance system characteristics or capabilities, different case definitions, type of pertussis confirmation tests used, public awareness of the disease, as well as real differences in the magnitude of the disease, or a combination of these factors. Our study highlights the need to standardize pertussis detection and confirmation in surveillance programs across Europe, complemented with carefully-designed seroprevalence studies using the same protocols and methodologies.

## Introduction

Pertussis (whooping cough) is a common, highly contagious and potentially serious acute respiratory disease that continues to be a major public health concern worldwide. The disease, mainly caused by *Bordetella pertussis* (*B*. *pertussis*), is transmitted to susceptible hosts directly via airborne respiratory aerosol droplets ejected from infected individuals while coughing [1]. The introduction of childhood vaccination in the industrialized world during the 1950s and 1960s led to a >90% reduction in the incidence and mortality of pertussis in those regions [2]. However, the number of reported pertussis cases has gradually increased in recent years. This resurgence of pertussis cases has been observed since the 1980s in a number of countries with high vaccine coverage rates in Europe and elsewhere, but this has by no means been a ubiquitous trend [3, 4].

In Europe, there is substantial variation in the reported incidence of pertussis between countries in any given year [5–9]—in 2012, the overall pertussis case rate was 10.9 cases per 100,000 population, with the highest rate reported in Norway (85.2 per 100,000) and the lowest with no reported cases was Malta [8]. Moreover, The Netherlands reported the highest total number of pertussis cases (N = 12,868), accounting for about 30% of the total reported to the European surveillance system by member states. Comparisons between countries are limited by differences in laboratory procedures, completeness of reporting, and differences in awareness of reporting of the disease.

The apparent resurgence of pertussis in some countries since the introduction of routine childhood vaccination appears to have altered the dynamics of the disease with a trend towards increased reports in older children, adolescents and adults [2, 5, 9–12], as well as an increase by an average of 1.3 years in the period between epidemics [13]. The reasons for the changes in disease dynamics are not well-understood [11, 12, 14]. However, acquired immunity following infection and vaccination wanes after 4–20 years and 4–12 years, respectively [15], and as such, epidemic cycles may be perpetuated by the continued transmission of pertussis among adolescents and adults. In addition, it has been suggested that the increased number of pertussis cases reported in adolescents and adults could have been due, in part, to the use of more sensitive detection methods and increased media awareness [4, 5, 11, 12, 14]. A number of other hypotheses have also been proposed including: antigenic divergence from vaccine strains; changes in vaccine composition or schedule (lower efficacy of acellular pertussis [aP] vaccines relative to whole cell pertussis [wP] vaccines or lower efficacy of pertussis vaccine associated with low coverage of the reinforcing dose between 12 and 24 months); tendency of acellular vaccines to induce Th2 rather than Th1 responses; population changes in immune profiles due to waning immunity and/or rates of natural boosting [4, 16, 17]. As such, due to the inherent complexities of pertussis disease dynamics, there is no widely accepted biological mechanism(s) to explain the current epidemiology of pertussis.

An overview of the changing incidence trends in a number of countries may help ascertain whether increased pertussis reports in some countries share common underlying drivers or whether there are specific features in each country. Here we report the results of an analysis of pertussis incidence in ten Central and Eastern European countries, based on information collected through an epidemiological survey. Understanding the underlying drivers of the changing pertussis epidemiology over time may help guide national immunization recommendations.

## Materials and Methods

This descriptive analysis of pertussis surveillance data pertains to information collected from 2000 to 2013 in ten Central and Eastern European countries: Bulgaria, the Czech Republic, Estonia, Hungary, Latvia, Lithuania, Poland, Romania, Serbia, and Slovakia. The current study was undertaken by an informal group of pertussis experts from Central and Eastern Europe who meet annually to discuss issues related to pertussis, and as such, the countries chosen correspond to the experts’ countries of origin. The analysis aimed to provide insights into the changing incidence of pertussis over time in a given country, and between countries.

### Data sources

Data were collated from relevant government institutions and national surveillance systems by investigators in their respective countries using a structured survey [18–31]. The survey focused on: the methods of pertussis surveillance (passive or active); case definition for pertussis (clinical case definition, laboratory confirmation protocols and methods); incidence and number of cases of pertussis by year, overall and by age group; population by year, overall and by age group; deaths due to pertussis; pertussis immunization schedule, modifications during the study period, and coverage (S1 and S2 Tables). Other validated sources or publications were also used where available to complete any missing information. No imputations were performed for missing data that could not be located.

### Data analysis

Data were entered into a Microsoft Excel spreadsheet and the annual incidence of pertussis per 100,000 population calculated by dividing the numbers of cases notified by population estimates obtained from national sources (for Lithuania [32], Latvia [24] and Romania [The National Institute of Statistics]) or directly from reported incidence rates from national surveillance systems. Descriptive statistics (medians, quartiles and interquartile range) were used to summarize the pertussis incidence rate over time, overall and by age-group. We assessed the changes in the pertussis incidence rates in each country, and the differences and similarities between countries in relation to a number of variables known or suspected to have an effect on pertussis incidence including vaccination program and surveillance system characteristics. Vaccination program variables included vaccine schedule, vaccine coverage and type of vaccine (wP or aP, vaccine switch). Surveillance system characteristics included type of surveillance (aggregate vs. case based reporting), targeted population (general population or part of the population, pediatric or sentinels network), clinical case definitions (European Centre for Disease Prevention and Control, World Health Organization or other), and methods and protocols for confirmation tests (culture, PCR, serology, other).

Finally, we appraised the relationship between the changing trends in reported annual pertussis incidence rates and the switch from wP to aP vaccination using the approach first developed by Hill [33]. Hill outlined a systematic approach to infer causation from statistical associations observed in epidemiologic data based on nine criteria: temporality, strength, consistency, specificity, dose-effect, plausibility, coherence, experiment and analogy. This approach defines and describes conditions that must be satisfied to pass from an observed association to the acceptability of a causal relationship. This framework for causal inference emerged in the 1950s and 1960s, following the international debate about the causal role of fat consumption on cardiac death rates, and later about the causal role of smoking in the occurrence of lung cancer. The "Hill Criteria" are considered more appropriate for analyzing observational data than tests of significance, especially when considering the impact of public health interventions [33–35]. The viewpoints or guidelines used to evaluate evidence have not changed since 1965 despite an increasing interest in causation and causal inference that has mainly evolved on conceptual issues required for estimation of causal inference. Currently, the nine viewpoints have been classified in two groups by the majority of epidemiologists [34]:

- one group, pooling 5 viewpoints (strength of association, consistency, specificity, temporality, biological gradient), summarizes data produced by the epidemiological studies (observational or experimental);
- the other group, pooling 4 viewpoints (plausibility, coherence, experiment, and analogy), summarizes data produced by non-epidemiologic studies and supporting causal hypothesis.

## Results

### Vaccine schedule, coverage and exposure

Infants in 9 of the ten countries received a 3-dose primary series before seven months of age, with a reinforcing dose between 12 and 36 months throughout the review period (Table 1). In Slovakia, infants continuously received the primary series with a 2+1 schedule. At the beginning of the review period, immunization started at 2 months (4 countries) or 3 months (6 countries), but two of them (Lithuania, Hungary) subsequently lowered the starting age from 3 to 2 months during the review period. Vaccination coverage with 4 doses (including the reinforcing dose of a 3+1 schedule) was ≥90% in all countries (excluding Lithuania and Slovakia) between 2000 and 2013, except Romania in 2009, 2011 and 2013 where the coverage was 82%, 89% and 88%, respectively. Coverage rates for 3 doses were also ≥90% throughout the study period in Lithuania (i.e., after dose 3 of a 3+1 schedule) and Slovakia (with a 2+1 schedule).

**Table 1:** Pertussis vaccination schedules by country.

Country	Time period	Primary series		Reinforcing/Booster doses						Coverage^∞^
				Toddlers		Pre-school age		Adolescence		(min–max)
**Bulgaria**	From 1992 to 2007	wP♦	2, 3, 4 months	wP♦	2 years					89.7–94.0%
	From 2008 to 2009	wP♦	2, 3, 4 months	aP	>16 months	aP	6 years			93.2–93.7%
	Since 2010	aP	2, 3, 4 months	aP	>16 months	aP	6 years			94.3% (2013)
**Czech Republic**	Until 2006	wP^□^	3, 4, 5 months	wP^□^	18–20 months	wP^□^	4–5 years			99.6–99.6%
	From 1 January 2007 to 14 February 2009	aP	3, 4, 5 months	aP	18 months	aP	5–6 years			92.8–99.3%
	Since 15 February 2009	aP	3, 4, 5 months	aP	18 months	aP	5–6 years	aP	10 years	92.1–98.0%
**Estonia**	From 2000 to 2007	wP^□^	3, 4.5, 6 months	wP^□^	2 years					91.7–95.8%
	Since 2008	aP	3, 4.5, 6 months	aP	2 years	aP	6–7 years	aP	15–16 years	96.0–96.7%
**Hungary**	Before 2006	wP♦	3, 4, 5 months	wP♦	3 years	wP♦	6 years			99.9–100%
	Since 2006	aP	2, 3, 4 months	aP	18 months	aP	6 years	aP	11 years	99.6–99.9%
**Latvia**	From 1958 to 2004	wP^□^	2, 4, 6 months	wP^□^	12–15 months					89.7–94.7%
	From 2005 to 2009	aP	2, 4, 6 months	aP	12–15 months					92.3–98.1%
	Since 2010	aP	2, 4, 6 months	aP	12–15 months	aP	7 years			90.0–97.9%
**Lithuania**	From 1961 to 2003	wP^□^	3, 4.5, 6 months	wP^□^	18 months					92.8–94.8%
	From 2004 to 2006	wP^□^	2, 4, 6 months	wP^□^	18 months					93.9–94.0%
	Since 2007	aP	2, 4, 6 months	aP	18 months	aP	6–7 years^§^			92.8–97.4%
**Poland**	From 1960 to 2002	wP^□^	2, 3, 5 months	wP^□^	16–18 months					94.7–94.8%
	Since 2003^#^	wP^□^	2, 3, 5 months	wP^□^	16–18 months	aP	6 years^‡^			94.7–96.0%
**Romania**	From 1961 to September 2008	wP♦	2, 4, 6 months	wP♦	12 months	wP♦	30–35 months			95.3–99.0%
	From 1 October 2008 to March 2009	aP	2, 4, 6 months	aP	13–15 months	aP	4 years			81.7–95.3%
	Since 1 April 2009	aP	2, 4, 6 months	aP	12 months	aP	4 years			81.7–93.8%
**Serbia**	Since 1960^†^	wP♦	2, 3.5, 5 months	wP♦	1–2 years					93.1–97.6%
**Slovakia**	From 2000 to 2006	wP^□^	3–4, 5–6, 11–12 months	wP^□^	3 years	wP^□^	6 years			98.5–99.4%
	From 2007 to 2008	aP	3–4, 5–6, 11–12 months	wP^□^	3 years	wP^□^	6 years			99.3–99.4%
	In 2009	aP	3–4, 5–6, 11–12 months			aP	6 years			99.2% (2009)
	Since 2010	aP	3–4, 5–6, 11–12 months			aP	6 years	aP	13 years	96.8–99.1%

aP, acellular pertussis vaccine (all aP used in the participating countries were produced by an international manufacturer); wP, whole-cell pertussis vaccine (^□^ wP produced by an international manufacturer and ♦ wP produced by local manufacturer)

^∞^ Coverage with 4 doses (including reinforcing dose) between 2000–2013 unless otherwise specified. Coverage data for 4 doses (including reinforcing dose) not available for Bulgaria in 2010–2012, for The Czech Republic in 2000–2003 and 2011–2012, and for Serbia in 2000–2004. Data for Estonia includes coverage up to age 10 years. Data for Lithuania and Slovakia are after dose 3 of a 3+1 schedule and 2+1 schedule, respectively.

^#^ No formal switch from wP in Poland, but aP widely available in private market

^†^ No formal switch from wP in Serbia, but aP widely available in private market

^‡^ aP booster introduced for 16-year-olds in 2015

^§^ aP booster introduced for 15–16-year-olds in 2016

At the start of the review period, Hungary, Slovakia, the Czech Republic and Romania had a preschool booster from about age 3 to 6 years, with Bulgaria, Estonia, Latvia, Lithuania and Poland introducing a preschool booster during the study period (Table 1). Serbia is the only country considered that does not have a preschool booster. Between 2006 and 2010, Estonia, Hungary, the Czech Republic and Slovakia introduced additional boosters for older children and adolescent.

All countries, except Poland and Serbia, switched to aP during the study period, with one or two countries switching each calendar year from 2005 to 2010. In six countries, all doses were switched from wP to aP simultaneously, while Bulgaria introduced aP for the pre-school booster dose in 2008 and for the primary series in 2010, and Slovakia introduced aP for primary series in 2007/2008 and for the pre-school booster in 2009. National immunization programs in Poland and Serbia are based on wP, but aP vaccines have been available on private market since 1999 and 2004, respectively, and in 2013 approximately 60% and 40% of infants in these two countries received aP vaccines (Aneta Nitsch-Osuch and Vladimir Petrović, personal communications).

### Surveillance network organization, clinical case definition and confirmation tests

Surveillance for pertussis is passive in all countries, with some differences in surveillance system characteristics, as well as clinical and laboratory diagnostic criteria (Table 2). In most countries, the clinical case definition used are those provided by the World Health Organization (WHO) [36] or the EU Decision 2119/98/EC of the European Centre for Disease Prevention and Control (ECDC) [37]. All countries changed their methods or protocols at least once in the study period. For example, Serbia did not use a clinical case definition or confirmatory tests until 2012. Bulgaria has used qualitative confirmation tests since 2000 and PCR was introduced in 2007 in the Sofia area only.

**Table 2:** Surveillance system, clinical and laboratory criteria used by country.

Country	Surveillance system	Clinical case definition	Microbiologic confirmation	Laboratory diagnosis		
				Culture	Serology	PCR
Bulgaria	Passive; Mandatory notification; Population-based surveillance; Aggregate reporting	WHO criteria	Yes^#^	Until 2008	≥ 1 change; Qualitative to quantitative tests (PHT & IF to ELISA); Serology assessment ended 2009	Since 2007^#^
Czech Republic	Passive; Mandatory notification; Population-based surveillance; Aggregate reporting	WHO criteria	Yes	Until 2000	≥ 1 change; Qualitative to quantitative test; kit change 2010	RT-PCR since 2009
Estonia	Passive; Mandatory notification; Population-based surveillance; Case-based reporting	WHO criteria	Yes	Rarely used	≥ 1 change; Qualitative to quantitative test	Since 2012, but rarely used
Hungary	Passive; Mandatory notification; Population-based surveillance; Case-based reporting	WHO/ECDC criteria	Yes	Until 2000	≥ 1 change; Qualitative to quantitative tests (hemagglutination to ELISA)	Since 2012
Latvia	Passive; Mandatory notification; Population-based surveillance; Case-based reporting	ECDC criteria	Yes	Rarely used	≥ 1 change; Qualitative to quantitative tests (PHT & WB to ELISA)	RT-PCR since 2012
Lithuania	Passive; Mandatory notification; Population-based surveillance; Case-based reporting	WHO/ECDC criteria	Yes	Until 2000	≥ 1 change; Qualitative to quantitative tests; Labsystem (2005–2010) & Euroimmun since 2010	Since 2010^‡^
Poland	Passive; Mandatory notification; Population-based surveillance; Case-based reporting	ECDC criteria	Yes	Rarely used	≥ 1 change; Qualitative to quantitative tests; introduced kits such as Novatec	Rarely used
Romania	Passive; Mandatory notification; Population-based surveillance; Case-based reporting	Reported cases diagnosed based on prolonged cough and a high level of WBC	Yes	Rarely used	≥ 1 change; No serology testing until 2008, then qualitative tests introduced	Sporadic since 2012
Serbia	Passive; Mandatory notification; Population-based surveillance (sentinel surveillance in one city [Novi Sad]); Case-based reporting	GPI clinical Case Definition used in 2012, in part, for sentinel surveillance in one city (Novi Sad)	Yes (since 2012)	Not used	≥ 1 change; Clinical case definition only until 2012, then quantitative tests introduced (Euroimmun)	Since 2012
Slovakia	Passive; Mandatory notification; Population-based surveillance; Case-based reporting	ECDC criteria	Yes	Until 2000	≥ 1 change; Qualitative to quantitative test	Since 2007

^#^ Sofia and surrounding regions only

^‡^ PCR is available only at the university hospitals and national reference laboratory

ECDC, European Centre for Disease Prevention and Control; ELISA, enzyme-linked immunosorbent assay; GPI, Global Pertussis Initiative; PHT, passive hemagglutination test; IF, immunofluorescence; RT-PCR, real-time polymerase chain reaction; WB, Western-blot; WHO, World Health Organization. The ECDC clinical case definition is that stipulated by EU Commission (8 August 2012) as follows [37]: Any person with a cough lasting at least two weeks and at least one of the following three symptoms: paroxysms of coughing; inspiratory "whooping", or post-tussive vomiting; or any person diagnosed as pertussis by a physician; or apnoeic episodes in infants. Laboratory criteria includes at least one of the following three criteria: isolation of *Bordetella pertussis* from a clinical specimen; detection of *Bordetella pertussis* nucleic acid in a clinical specimen; or *Bordetella pertussis* specific antibody response. Serology results need to be interpreted according to the vaccination status. The WHO clinical case definition is as follows [36]: A case diagnosed as pertussis by a physician or any person with a cough lasting at least two weeks with at least one of the following symptoms: paroxysms (i.e. fits) of coughing; inspiratory whooping; post-tussive vomiting (i.e. vomiting immediately after coughing) without other apparent cause. Laboratory confirmation includes: isolation of *Bordetella pertussis*; detection of genomic sequences by means of polymerase chain reaction; or positive paired serology. nucleic acid. The GPI clinical case definition for surveillance purposes is dependent on the age of the person [38]. For those aged 0–3 months, this includes cough and coryza or minimal fever plus: whoop, apnea, post-tussive emesis, cyanosis, seizure, pneumonia; or close exposure to an adolescent or adult family member with a prolonged afebrile cough illness. For those aged 4 months to 9 years, this includes paroxysmal cough with no or minimal fever plus: whoop, apnea, post-tussive emesis, seizure, worsening of symptoms at night, pneumonia; or close exposure to an adolescent or adult family member with a prolonged afebrile cough illness. For those aged ≥10 years, this includes non-productive, paroxysmal cough ≥2 week duration without fever plus: whoop, apnea, sweating episodes between paroxysms, post-tussive emesis, or worsening of symptoms at night. Laboratory confirmation in individual suspected cases includes: detection by polymerase chain reaction or isolation of *Bordetella pertussis* in those aged 0–3 months, and increased white blood cell count (≥20,000 leukocytes/≥ 10,000 lymphocytes) if the cough is <3 weeks duration. For those aged 4 month–9 year and those aged ≥10 years, a positive PT-IgG antibody response if ≥ 1 year after vaccination or detection of *Bordetella pertussis* by polymerase chain reaction if the cough is <3 weeks duration confirm pertussis.

The majority of the countries notified pertussis cases based on clinical case definitions and confirmatory tests with qualitative serology or culture only before 2005. However, qualitative serology tests have since been gradually replaced with more sensitive and specific quantitative serology tests. Detection of *B*. *pertussis* nucleic acid by polymerase chain reaction (PCR), including real-time (RT)-PCR, was introduced between 2003 and 2012. By 2012, nearly all the countries used PCR and/or serological ELISA tests (in house or commercial kits) to confirm the majority of cases [39, 40].

### Epidemiological changes

#### Intracountry changes

**Fig 1:**
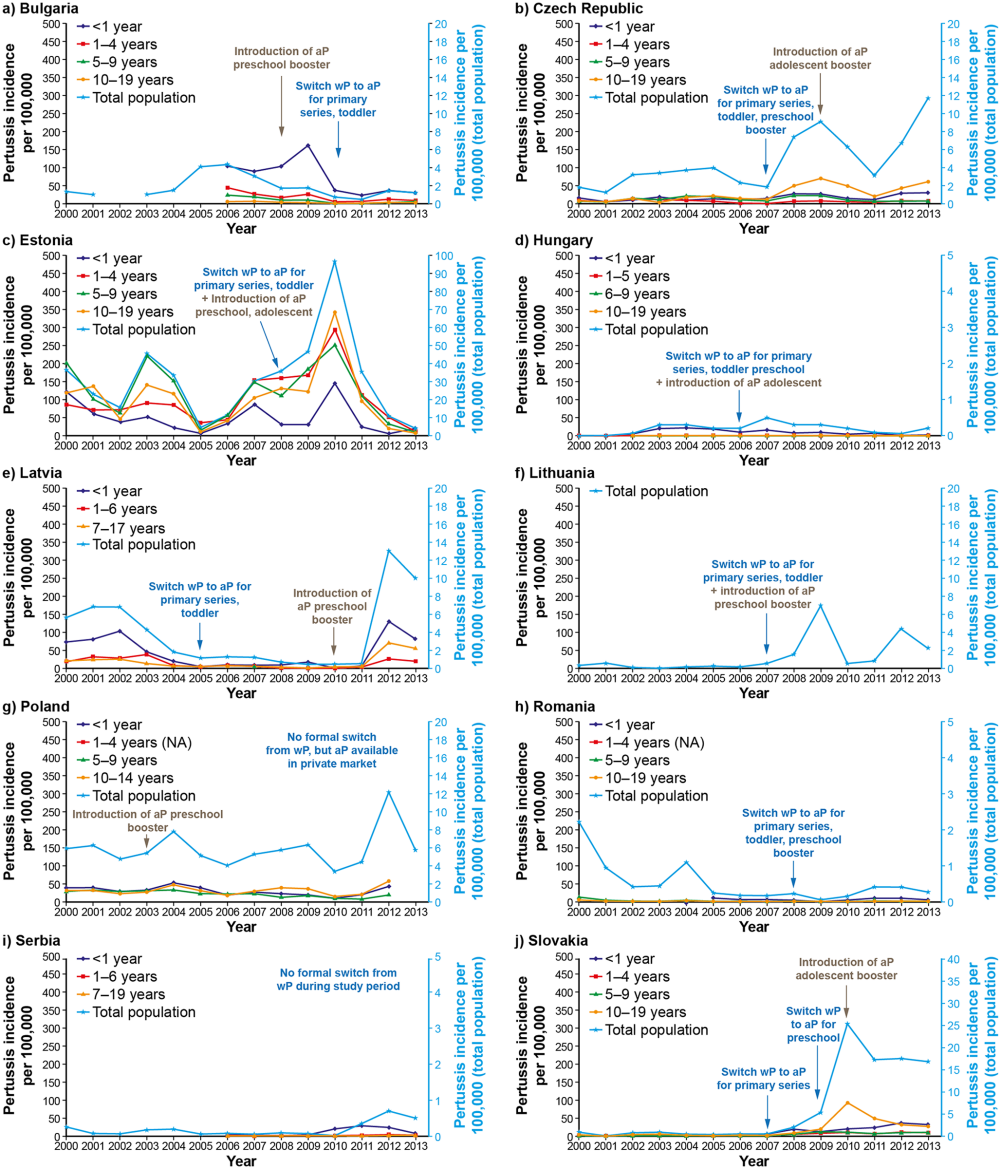
Pertussis incidence in the ten participating Central and Eastern European countries, 2000–2013. aP: acellular pertussis vaccine combined with diphtheria and tetanus toxoids. wP; whole cell pertussis vaccine combined with diphtheria and tetanus toxoids. Data not available by age group for Lithuania.

Bulgaria: The annual reported pertussis incidence ranged from 0.5 to 4.4 per 100, 000 population (Fig 1a). The introduction of the aP preschool booster vaccination in 2008 and the switch from wP to aP vaccines for the primary and toddler vaccinations in 2010 occurred at a time when the year-on-year reported incidence of pertussis had been generally decreasing. The introduction of PCR in 2007 (in the Sofia area) and the evolution of the serology tests from 2006 to 2009 (Table 2), moving from passive hemagglutination test, immunofluorescence and culture to PCR and more quantitative serological ELISA tests, also appear to coincide with a decreasing trend in the incidence of reported pertussis. The overall age-group trend in pertussis incidence rates (data available between 2006 and 2013 only) is suggestive of a decrease in reported cases over time across all age groups.

Czech Republic: The annual reported pertussis incidence ranged from 1.2 to 11.7 per 100,000 population (Fig 1b). The overall trend was suggestive of a gradual underlying increased rate in pertussis notifications during the study period. The switch to aP for the primary series, for the reinforcing dose in the second year of life, and for the preschool booster in 2007 was followed by a sharp increase in the reported incidence of pertussis. However, there was a reduction in the two years following the introduction of the aP adolescent booster vaccination (at 10–11 years of age) in 2009 before the incidence rate started to increase again. New serological assay kits and RT-PCR were introduced for the diagnosis of pertussis around 2010 (Table 2), but these changes do not to appear to have affected the underlying trend in the incidence of reported pertussis. Since 2008, adolescents (10–19 year age group) have had the highest reported incidence of pertussis.

Estonia: Estonia was the country with the highest reported incidence of pertussis cases among the ten participating countries, with annual incidence rates that ranged from 4.1 to 96.9 per 100,000 population (Fig 1c). The switch to aP vaccines and the concomitant introduction of a preschool and an adolescent booster in 2008 was associated with an increase in the reported incidence of pertussis—however, the reported pertussis incidence had started to gradually increase two years earlier and continued to do so for another two years after the introduction of aP vaccines (peaking at 96.9 per 100,000). No temporal association with vaccines switch and the introduction of boosters was observed. For most of the study period, pertussis was confirmed using qualitative serological tests, before quantitative ELISA tests were introduced in 2010/2011. Incidentally, the highest incidence of pertussis was reported 2010 but this returned to similar levels as before in 2011. In general, the trends in the reported incidence of pertussis were similar across all age groups, but tended to be lowest in infants.

Hungary: Hungary, along with Serbia (see below), had the lowest reported annual incidence of pertussis among the ten countries: incidence rates ranged from 0.01 to 0.5 per 100,000 population (Fig 1d). The switch to aP vaccines for the primary series of vaccination and for toddlers, and the concomitant introduction of a preschool and an adolescent booster (at age 11 years) with aP vaccines in 2006 was not associated with an immediate decrease in the reported incidence of pertussis. There was a change in the methods used to diagnose pertussis in 2008, moving from passive hemagglutination tests to quantitative ELISA tests and PCR (Table 2). These changes in diagnostic methods were followed by reduced incidence of pertussis reported in the subsequent years. The incidence of pertussis was highest in infants. There were too few cases reported or data were missing for the other age groups to observe any meaningful trends.

Latvia: Approximately ten-fold decrease in the reported annual pertussis incidence rate was noted between 2000 and 2011, before a dramatic increase in 2012 (Fig 1e). Over the period, the annual incidence of reported pertussis cases ranged from 0.4 to 13 cases per 100,000 population. The switch to aP vaccines for the primary vaccination series and for the reinforcing dose at 12–15 months of age in 2005 did not appear to modify the underlying decreasing trend in incidence of reported pertussis cases. The introduction of a preschool booster (at 7 years of age) in 2010 was followed two years later by an increase in reported cases. The incidence of pertussis was highest in infants throughout the study period.

Lithuania: The annual reported pertussis incidence ranged from 0.01 to 6.96 per 100,000 population (Fig 1f). In the seven years prior to the switch to aP vaccines for the primary series of vaccination and for the reinforcing dose at 18 months of age, and concomitant introduction of a preschool booster (at 6–7 years of age) in 2007, the average incidence of pertussis ranged from 0.01 to 0.5 per 100,000 population. The switch to aP vaccines was accompanied by an increase in reported pertussis incidence that peaked in 2009, and subsequently greater fluctuation in the reported rates than were seen in the earlier years of the study. The laboratory methods used to diagnose pertussis changed to quantitative ELISA tests and included PCR around 2010 (Table 2). It is not clear how these changes in laboratory methodology affected pertussis reporting rates. Data on the incidence of pertussis by age group was not available.

Poland: The annual incidence of reported pertussis cases ranged from 3.3 to 12.2 per 100000 population (Fig 1g). The introduction of aP vaccines for the preschool booster (at 6 years of age) in 2003 did not appear to have modified the underlying trend in the fluctuating incidence of reported cases. Poland is one of two countries (the other is Serbia, see below) that did not switch the primary series of vaccination or reinforcing dose to aP during the study period. The vast majority of notified pertussis cases were confirmed by serology and rarely by PCR or culture up to 2012. It is not clear whether changes in laboratory diagnosis during that time played a role in the reported incidence of pertussis, and in particular in the doubling of the reported incidence of pertussis in 2012 compared to other years. The incidence of reported pertussis tended to be highest in infants up until 2006, but since then, the highest rates have consistently been reported in adolescents (10–14 years of age).

Romania: Between 2000 and 2008 (the year of switch to aP vaccines for the primary series of vaccination, for the reinforcing dose, and for preschool booster), there was an underlying reduction in incidence of reported pertussis cases from 2.2 to a minimum of 0.22 per 100,000 population (Fig 1h). Although there was reduction in reported pertussis cases in 2009 (the year after switching to aP) to 0.5 per 100,000 population, this was not sustained with the incidence returning to similar levels as before. PCR diagnosis of pertussis was introduced in 2012 (Table 1), but used only sporadically. Although the data are incomplete for the age groups assessment, infants tended to have the highest rates reported where comparative data were available.

Serbia: Like Hungary, Serbia had the lowest reported annual incidence of pertussis among the ten countries, with incidence rates that ranged from 0.01 to 0.7 per 100,000 population (Fig 1i). Serbia (like Poland) did not switch the primary series of vaccination or toddler boosters to aP during the study period. It is possible that the introduction of quantitative ELISA tests and RT-PCR for the diagnosis of pertussis in 2012 may have contributed to the higher rates of pertussis reported that year. The incidence of pertussis was highest in infants in the years where age-group data were available.

Slovakia: The annual reported pertussis incidence ranged from 0.01 to 0.8 per 100,000 population in the years preceding the switch to aP vaccines for the primary series of vaccination in 2007 (Fig 1j). From 2008, an increase in the incidence was observed, but it was mainly in the 10–19 year age group vaccinated with wP. The incidence eventually peaked in 2010, the year of introduction of an aP adolescent booster (at 13 years of age). The annual incidence of reported pertussis declined in the subsequent year and appears to have stabilized at about 17 per 100,000 population in the later part of the study, a level that is much higher than reported earlier. It is possible that the introduction of PCR for the diagnosis of pertussis in 2007 and quantitative ELISA test from 2010 (Table 2) may have had a role in the increased incidence of reported pertussis in the subsequent years. Interestingly, the year the adolescent booster was introduced was also the year that the adolescent group (10–19 years) had the highest reported incidence of pertussis. Subsequently, similar annual rates of pertussis have been reported in the adolescent group and in infants.

#### Intercountry changes

**Fig 2:**
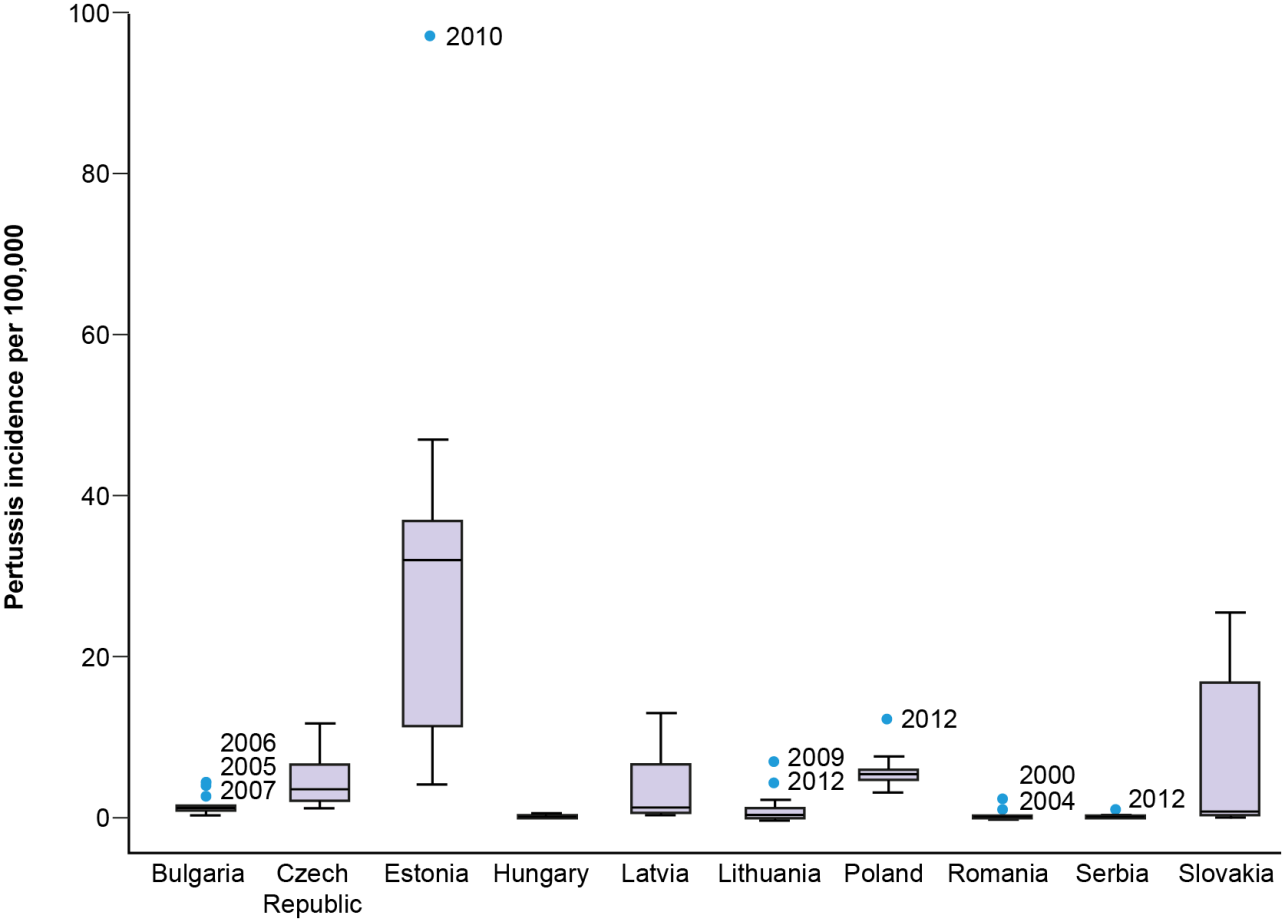
Boxplot of overall annual pertussis incidence rates between 2000 and 2013 for each of the ten participating Central and Eastern European countries. Each boxplot represents the median (black line in box) and the 25th and 75th percentile incidence rates (edges of the box). The whiskers represent 1.5 x inter-quartile range. Outliers are represented as blue dots.

**Fig 3:**
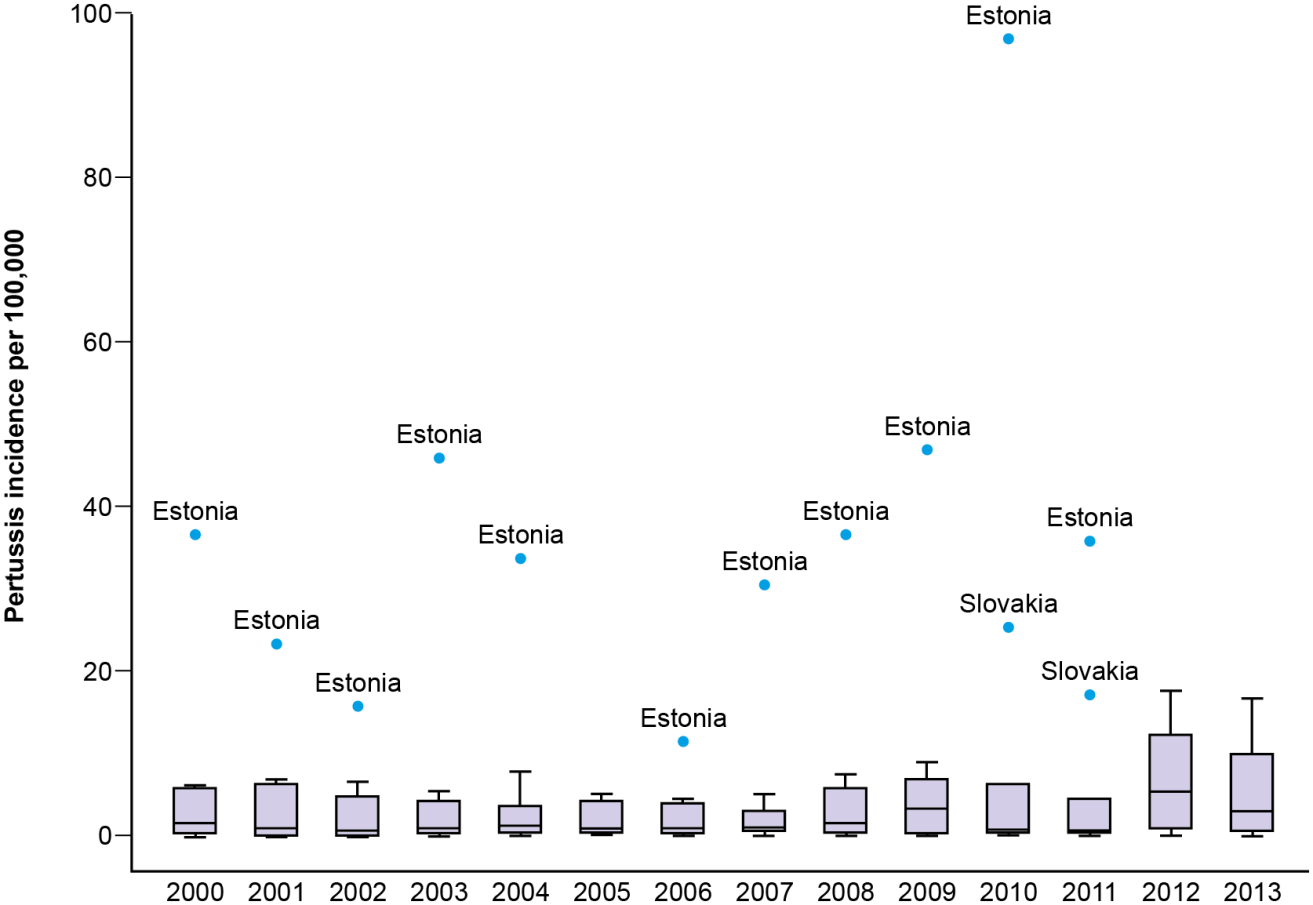
Boxplot of the overall annual incidence of pertussis reported across all participating countries for each of the calendar years in the study period. Each boxplot represents the median (black line in box) and the 25th and 75th percentile incidence rates (edges of the box). The whiskers represent 1.5 x inter-quartile range. Outliers are represented as blue dots.

The annual notification rates of pertussis and general trends observed during the study in the ten participating countries are summarized in Fig 2 —this figure displays the same time series data presented in Fig 1 in a boxplot of annual incidence rates by country. There were wide disparities in the annual reported pertussis incidences across the ten countries, ranging from 0.01 to 96.9 per 100,000 population. Estonia had the highest annual incidence of reported cases. Overall, the reported pertussis incidence rates across countries were highly heterogeneous (Fig 3). Moreover, except in 2012 with five countries experiencing an incidence peak, we did not observe synchrony in the reported pertussis incidence rates between countries during the study period.

There were 11 pertussis associated deaths reported across the ten countries during the study period, most of which occurred in unimmunized infants (Table 3); the case fatality rate over the study period ranged from 0.06 to 1.5 per 1000 cases in the six countries that reported deaths.

**Table 3:** Pertussis associated deaths in 10 Central and Eastern European countries (2000–2013).

Country	Number of deaths	Year	Age	Gender	Vaccination status	Circumstances of death
**Bulgaria**	0					
**Czech Republic**	4	2005	Newborn	Male	Unvaccinated	Too young for vaccination.
		2007	4 months	Female	Unvaccinated	Vaccination postponed because of acute respiratory illness (later confirmed as the first signs of pertussis disease).
		2008	4 weeks	Female	Unvaccinated	Too young for vaccination.
		2009	2 months	Female	Unvaccinated	Too young for vaccination.
**Hungary**	0					
**Estonia**	1	2007	1 month	Male	Unvaccinated	Family history: pertussis was diagnosed in mother and older brother before infant.
**Latvia**	2	2012	1 month	Female	Unvaccinated	Mother was not vaccinated during pregnancy. Cause of death: ARDS, MODS, hyperleukocytosis, septic shock, right lower extremity necrosis, left foot ischemia. Pertussis DNA positive.
		2013	9 months	Female	Unvaccinated	Refusal of vaccination form signed by parents. Cause of death: brain edema, pulmonary emphysema, atelectasis, pulmonary hemorrhage. Pertussis DNA positive.
**Lithuania**	1	2012	5 months	Male	Unvaccinated	Child from a ‘natural life style believer’ family. No vaccination, no other medical services. Child started coughing about a month before admission to the hospital. No treatment at home except ‘natural medicines’. Seizures progressed despite treatment: coma, bradycardia. Death after 32 hours of treatment.
**Poland**	2	2005	5 months	Female	Unvaccinated	Premature delivery (32 weeks gestation). Reason for lack of vaccination: neurological contraindications
		2008	68 years	Male	Unvaccinated	Cause of death: cardiopulmonary insufficiency
**Romania**	0					
**Serbia**	0					
**Slovakia**	1	2013	57 years	Male	Unknown	Underlying chronic pulmonary disease and cor pulmonale

ARDS, acute respiratory distress syndrome; DNA, deoxyribonucleic acid; MODS, Multi organ dysfunction syndrome

### Hill criteria for causality

In this section, we briefly appraise the relationship between the switch from wP to aP vaccination and the changing trends in reported annual pertussis incidence rates using Hill’s criteria of causality. The idea of these causality considerations is to provide a mechanism for inferring causal association between two factors before it is possible to assume causality from multiple information sources rather than a binary yes or no approach to causality [33, 34]:

- *Temporal sequence—not supported*. There was no temporal sequence observed in the switch from wP to aP and reported pertussis incidence rates in all the eight countries that switched to aP during the study period. Although it could be loosely argued there were increased pertussis incidence rates after the switch to aP in six countries, the time period between the switch and the rising incidence of notified pertussis varied from four years before the switch to seven years after. In addition, similar fluctuating trends were also observed in two countries where wP was mainly used throughout the study period.
- *Consistency*—*not supported*. There was no consistent pattern observed in reported pertussis incidence rates following the switch from wP to aP in the eight countries that switched to aP during the study period. Epidemiologic trends of increased pertussis incidence rates following widespread aP use reported from observational studies undertaken in Australia and the USA have been proposed as evidence of a switch effect [41–45]. However, there are also reports of increased pertussis incidence rates in countries that continue to rely on wP [46–48]. In addition to observations from our analysis, this leads us to conclude that the evidence of a switch effect is inconsistent.
- *Strength of association—not supported*. Due to the low consistency and the variable temporal sequence, the strength of this association was difficult to estimate. Using the median value as reference by country, in the six countries showing some sort of temporal sequence the highest reported incidences after the switch corresponded to 1.2- to 10-fold increase in the overall incidence. However, in the two countries (Bulgaria and Romania) where it could be argued that there was no temporal sequence with the switch to aP, and in the two countries (Poland and Serbia) that did not switch to aP on a national level, the variation of the notified overall incidences between median value and maximal values was similar with a range from 1.2- to 14-fold increase towards the end of the study period.
- *Specificity—not supported*. Exposure to aP was not universally linked to increased incidence of pertussis. In each country, among the different age groups exposed to wP or aP, the variation of pertussis incidence was similar and not specific to age groups exposed to aP. In addition, the reports of increased incidence in countries where wP vaccines remain the standard of care argue against the specificity of the association [46–48].
- *Dose-effect response—not supported*. The present review did not provide an opportunity to assess this aspect of the association. The effect of the number of wP priming doses on the possible association with pertussis incidence has been studied in the US and Australian settings [42, 49]. However, whether there is a possible association remains controversial, since potential confounding factors were identified in the database used for the US study [42] and the association lacked statistical significance in the Australian analysis [49].
- *Biologic plausibility—inconclusive*. A number of plausible mechanisms to explain a possible switch effect have been postulated and include faster waning of vaccine-induced immunity, differential orientation of the immune response (Th1 and Th17 vs. Th2) and changes in characteristics of circulating *B*. *pertussis* strains under selective pressure from vaccination [12]. However, these hypotheses have been based on studies, some of which suffered from limitations related to statistical power, potential confounding or were conducted in animals without human-study confirmation. In contrast, human epidemiologic modelling suggested otherwise [50].
- *Coherence—not supported*. Although the effect of the switch from wP to aP for the primary immunization series has been proposed as a cause for the resurgence of pertussis in some countries, alternative hypotheses have also been put forward to explain the reported variations of pertussis incidence over time such as, improvement in surveillance methods [51], changes in disease circulation patterns [52], suboptimal vaccination coverage [53], or change of disease awareness [51, 54], leading to a broader consensus that the resurgence of pertussis is likely the result of a multifactorial effect.
- *Experimental evidence—inconclusive*. Some of the randomized vaccine efficacy trials in the 1990s showed that wP vaccines had higher efficacy estimates than aP vaccines [11]. Experimental data in baboons and mice have also suggested that the immunity elicited by wP vaccines better protects against colonization and transmission than immunity elicited by aP vaccines [55, 56], and thus, may provide a rationale for pertussis resurgence. However, these studies in animals were not statistically powered to confirm any difference between treatment groups and it remains to be determined whether these observations are applicable to humans [50].
- *Analogy—not supported*. We are unaware of similar effects with other vaccines, so this Hill criterion is neither refuted nor supported.

In summary, the analysis of the possible causal association of the switch from wP to aP vaccine with a possible resurgence of pertussis using the Hill criteria has lead us to conclude that the evidence from our study is not supportive of such a causal association. Expanding the evidence review to additional published data provides some support, but with a more complex picture of possible causes for pertussis resurgence, of which the contribution of the wP to aP vaccine switch remains inconclusive. Finally, based on the evidence from the Australian or US settings, any effect the switch between vaccines may have had on pertussis epidemiologic trends would likely be undetectable given that the period of switch to aP vaccine assessed in our study was less than 10 years in most of the countries.

## Discussion

This article provides a comprehensive analysis of the annual incidence of pertussis collected in ten Central and Eastern European countries from 2000 to 2013. The epidemiological data collected show that pertussis continues to be a relevant public-health concern despite high vaccination coverage across these countries. There was heterogeneity in the reported annual incidence rates and trends observed across countries. The greatest burden appears for the most part in infants in Bulgaria, Hungary, Latvia, Romania, and Serbia, but not in the other participating countries where the burden may have shifted to older children, and adolescents. Similar heterogeneity in pertussis incidence rates (including wide differences in rates between countries) and trends have been reported previously in five of the countries included in our study [57], across Europe [6–8] and globally [4]. The heterogeneity in reported incidence rates may be related to a number of factors including surveillance system characteristics or capabilities, different case definitions, type of pertussis confirmation tests used, public awareness of the disease, compliance with reporting, as well as real differences in the magnitude of the disease, or a combination of these factors.

There were very few pertussis-associated deaths reported during the study period; the case fatality rate ranged from 0.06 to 1.5 per 1000 cases in those countries that reported deaths. This low mortality rate is consistent with that published in a EUVAC.NET surveillance report for the period 2003–2007; during that period there were 27 pertussis-associated deaths reported across eight countries (including four countries participating in our study) resulting in a case fatality rate ranging from 0.1–7.1 per 1000 cases in those countries [58]. The case fatality rates reported in our study and by EUVAC are based on very few deaths and likely represent a gross underestimation. In general, deaths due to pertussis are not well recognized.

Differential underreporting between countries are common problems with all surveillance systems and may have contributed to the observed heterogeneity in the incidence of pertussis reported. Under-detection in adolescents and adults may be a particular problem as the disease may not be recognized—a prolonged cough illness might be the only clinical feature in this population. Indeed, data was lacking for the incidence of pertussis reported in adults. The extent of underreporting in the countries included in the current study is difficult to ascertain but may be substantial for some. For example, a study at a single hospital in Bucharest, Romania, assessing the circulation of *B*. *pertussis* in the locality in 2012 identified 51 suspected cases of which 32 (63%) were confirmed using culture, real-time PCR, and in house ELISA methods. Of these cases, the hospital sent 46 samples to the National Reference Laboratory, whereas only 251 samples were referred by the whole country during that year. The number of cases confirmed by the hospital represented about half of the total (32/70) confirmed cases in the country, but these 32 cases were not entered into the national surveillance system, as they were detected through a separate project [59]. Underreporting likely occurs even in countries with high incidences of reported cases. Prospective studies assessing the incidence of pertussis in adolescents and adults have reported values of 370 and 500 per 100,000, respectively [60, 61]. As such, it may be argued that the highest incidence rates in our study are closest to the real incidence of pertussis. Moreover, in Estonia, a cross-sectional serosurvey of 9- to 14-year-olds undertaken between April–August 2012 estimated the incidence of *B*. *pertussis* infection to be 6.3%, which was >60-fold higher than officially reported [62]. However, *B*. *pertussis* infection is frequently asymptomatic and its incidence therefore is much higher than that of pertussis disease [63]. A recent review of pertussis seroprevalence studies confirms the gross underestimation of pertussis incidence based on reported cases compared to findings from seroprevalence studies [64].

During the study period, a number of changes in immunization schedules occurred which may have played a part in the changing incidence of pertussis reported. These changes include: earlier first dose immunization, later preschool administration, and preschool and adolescent booster introduction. The changes in vaccine immunization schedules were generally introduced to provide direct and indirect protection for infants who are too young to be fully immunized from severe disease, and as such, would likely have affected the age-stratified disease incidence. The introduction of the preschool and/or adolescent boosters have been suggested to possibly reduce transmission from older siblings to young infants not or not fully vaccinated [65, 66], but others have reported that this effect may be modest at best or not observed [67–69]. In contrast, a modelling study has also suggested that increased vaccination of children would cause a shift in age-specific incidence towards an increased number of cases reported in teenagers and adults [70].

Eight of the ten countries in our study switched from wP to aP vaccines between 2005 and 2010, but two countries continued their national infant immunization programmes with wP vaccine (Poland and Serbia). For the countries that switched to aP, three distinct time periods can be identified: 1) before 2005 where all countries used wP; 2) between 2005 and 2010 where a mixture of both wP and aP were in use, with differing exposure rates to wP and aP by age-group within each country; and 3) after 2010 when all new birth cohorts were exposed to aP vaccines. Since 2010, progressively, all children younger than 3 years should have been exposed to aP (excluding Poland and Serbia). Over the survey period, all children older than 7 years would have been exposed to wP at least for their primary series and reinforcing dose, and all the population in Serbia and Poland would have been exposed to wP vaccines (although aP has been available in the private market). With the current hypothesis of reduced effectiveness and faster waning immunity with aP than wP vaccines, a switch effect would likely be observed in children younger than 7 years based on differential priming effect of aP and wP in those children who did not received a wP primary series. Latvia may have had a temporal association with the switch from wP to aP, with an increased incidence in reported cases occurring 7 years after the switch to aP for the primary series and reinforcing doses. However, in Latvia, as in other countries, the increasing incidences were reported concomitantly in age groups exposed to aP (mainly infants) and to wP (7–17 year olds). Our observations do not support a switch effect from wP to aP during the study period, but it is possible that the time period since switching in most countries in our study was too short to observe such an effect that has been described more than 10 years after switching in the USA [71].

The wide variation in pertussis incidence reported between countries may also be related to the difference in the procedures and laboratory methods used to confirm *B*. *pertussis* infection. This information was difficult to collect precisely and introduced a potential bias in our analysis. The heterogeneity in methods used for the laboratory confirmation of pertussis diagnosis among European countries has been previously highlighted as a limiting factor in evaluation of the effects of different pertussis immunisation programmes across Europe [39, 40]. In each country, the confirmation tests used in routine practice have been modified at least once over the study period. Recommendations for standardization and harmonisation of serological tests and nucleic acid amplification tests for the diagnosis of *B*.*pertussis* infections in Europe have since been published [72, 73]. Moreover, nine of the ten countries in our study (excluding Serbia) were involved in surveys and external quality assurance (EQA) programs implemented in Europe since 2011 to standardize laboratory methodologies and protocols to ensure accurate and consistent diagnosis of pertussis reported to national surveillance systems [39, 40].

These on-going initiatives may have influenced the incidence of notified pertussis cases after 2011, and could explain, at least partially, the increase in cases reported in 2012 including the two countries where most infants were exposed to wP throughout the study period. Increased laboratory testing in suspected cases and the more widespread use of enhanced sensitivity PCR diagnostic methods may have contributed to the increasing incidence of reported cases of pertussis [3, 51]. For example, in Serbia, we observed an increase in the incidence of reported pertussis in 2012, the year after starting confirmation of pertussis cases with serology and PCR in the sentinel network. In addition, the increase in the reported incidence in Latvia in 2012 occurred in parallel with the introduction of quantitative ELISA tests and RT-PCR in 2011/2012. It is also possible that increased physician awareness as a result of publicity surrounding the on-going pertussis initiatives may have increased reporting rates. There are examples of increased media attention or medical information campaigns drawing awareness to pertussis resulting in increased reporting rates to national surveillance systems [74]. Greater physicians awareness of pertussis has been suggested by the WHO SAGE pertussis working group to have, in part, contributed to the increase in cases reported in recent years in the majority of countries where such increases have been noted [3]. It is possible that physician awareness may have changed during the survey period, however, it is difficult to assess awareness of pertussis among reporting physicians and such studies are lacking.

The variation in incidence of pertussis infection with age is well-described and is one of the main changes reported in the vaccine era with increasing incidence in infants, especially those too young to be immunized or partially immunized, and in adolescents and adults population in Europe [6] and in north America [12]. Of note, the majority of deaths reported in our study were of infants who had either not been vaccinated or were too young to have received the full series of primary vaccinations. Age can be also a confounding factor in assessing the relationship between aP switch and incidence of pertussis due to the strong co-linearity between age and type of vaccines in observational surveys investigating the effect of the switch from wP to aP [75].

This review of the reported incidence of pertussis in ten Central and Eastern European countries has several limitations. We did not attempt to document hospitalisations associated with pertussis. In addition, we did not sufficiently document the changes in laboratory methods for the diagnoses of pertussis over time and between countries to determine whether increased laboratory testing and/or use of more sensitive methods may have influenced pertussis reporting rates particularly in more recent years. Although it is likely that increased physician awareness may have contributed to the increased incidence of pertussis reported, this would have been difficult to document. These two issues would have been sufficient to confound any assessment of the role of vaccine related factors in the changing incidence of reported pertussis. Nonetheless, our analysis provides a reasonable approach towards a greater understanding of pertussis epidemiology at the global level.

## Conclusions

The annual reported rates of pertussis and general trends observed during the study period across the ten participating countries were heterogeneous, with wide disparities in the incidences of reported cases. There was no clear cycle or obvious synchrony in reporting rates using overall or infant incidence data across the countries, except during 2012 where it could be argued that peak incidence rates were reported in five countries. There was no consistent pattern associated with the switch from wP to aP vaccines on reported pertussis incidence rates. The heterogeneity in reported data probably does not reflect significant true differences in pertussis incidence but is rather related to a number of factors including surveillance system characteristics or capabilities, different case definitions, sensitivity of pertussis confirmation tests used, awareness of the disease, as well as real differences in the magnitude of the disease, or a combination of these factors. These data confirm the necessity to standardize pertussis surveillance programs in Europe, especially for the detection and confirmation of pertussis cases and to complete this approach with carefully-designed seroprevalence studies using the same protocols and methodologies. This will allow for more reliable inter-country comparisons of the magnitude of *B*. *pertussis* circulation and disease.

## Supporting Information

S1 Table Pertussis epidemiology and vaccine recommendations in Central and Eastern European countries questionnaire. (DOC)

S2 Table Pertussis in Central and Eastern European countries follow-up questionnaire. (DOC)
