# Supplementary material for: Comparative Epidemiologic Characteristics of Pertussis in 10 Central and Eastern European Countries, 2000-2013
Source: PLoS One. 2016 Jun 3;11(6):e0155949. doi: 10.1371/journal.pone.0155949 (PMC4892528; doi:10.1371/journal.pone.0155949)
Supplement: S1 Table — (DOC) [file pone.0155949.s001.doc]

Table S1: REVIEW OF PERTUSSIS EPIDEMIOLOGY AND VACCINE RECOMMENDATIONS IN CENTRAL AND EASTERN EUROPEAN COUNTRIES.

Please provide the following information for this review manuscript.

COUNTRY:

AUTHOR:

| **Variable** | **Information/response** | **Source/reference*** |
| --- | --- | --- |
| Surveillance data available? Yes/no. |  |  |
| If yes: |  |  |
| 2000-present day? Yes/no.  - If no, surveillance start date |  |  |
| Is the surveillance active or passive? |  |  |
| Age range(s) under surveillance (if applicable) |  |  |
| Entire country or specific regions included in surveillance?  - If not entire country, specify regions covered |  |  |
| Please provide separately in **table form as original data** (e.g. not simply graphs) and any relevant publications |  |  |
| Clinical case definition  - Yes/no   - If yes, provide definition |  |  |
| Laboratory case confirmation   - Yes/no - If yes, analysis method used (PCR, serology, or culture) |  |  |
| Immunisation schedule   - Provide history of primary series and booster vaccination schedules, including dates and schedule changes - aP or wP (including if there has been a switch, and if so, when) |  |  |
| Coverage (% population) (include time period if relevant)  - Primary series  - Boosters  - Toddler  - Pre-school  - Adolescent |  |  |

**whenever possible the source of the information must be given*
