# Supplementary material for: Comparative Epidemiologic Characteristics of Pertussis in 10 Central and Eastern European Countries, 2000-2013
Source: PLoS One. 2016 Jun 3;11(6):e0155949. doi: 10.1371/journal.pone.0155949 (PMC4892528; doi:10.1371/journal.pone.0155949)
Supplement: S2 Table — (DOC) [file pone.0155949.s002.doc]

**Table S2: CEEPAG Publication**

**Additional data to be collected if available**

**2013 data**

*Please provide available data for 2013 (if not available, please specify “not available”)*

| **Number of cases in 2013** |  | | | |
| --- | --- | --- | --- | --- |
| **Overall incidence in 2013** |  | | | |
| **Data by age group** | **Age group** | **Number of cases** | | **Incidence** |
|  |  |  |  |  |
|  |  |  |  |  |
|  |  |  |  |  |
|  |  |  |  |  |
|  |  |  |  |  |
| **Death**  **If death case, please specify age, vaccination status, date since last pertussis dose and confirmation method (Culture, PCR or serology)** |  | | | |
| **Vaccine coverage rate** | **DTP3 primary series** | |  | |
|  | **Toddlers 2^nd^ year** | |  | |
| **Change in clinical case definition** |  | | | |
| **Change in surveillance method** |  | | | |
| **Change in immunization schedule** |  | | | |

**Surveillance methods**

*Please provide available data for 2000-2013 (if not available, please specify “not available”)*

Serology

|  | **Did the laboratories use serology to confirm suspected cases? (Yes/No)** | **If yes, which method(s)** | **Number of cases confirmed by serology** | **% of cases confirmed by serology (among confirmed cases)** |
| --- | --- | --- | --- | --- |
| **2000** |  |  |  |  |
| **2001** |  |  |  |  |
| **2002** |  |  |  |  |
| **2003** |  |  |  |  |
| **2004** |  |  |  |  |
| **2005** |  |  |  |  |
| **2006** |  |  |  |  |
| **2007** |  |  |  |  |
| **2008** |  |  |  |  |
| **2009** |  |  |  |  |
| **2010** |  |  |  |  |
| **2011** |  |  |  |  |
| **2012** |  |  |  |  |
| **2013** |  |  |  |  |

**PCR**

|  | **Did the laboratories use PCR to confirm suspected cases? (Yes/No)** | **If yes, which method(s)** | **Number of cases confirmed by PCR** | **% of cases confirmed by PCR (among confirmed cases)** |
| --- | --- | --- | --- | --- |
| **2000** |  |  |  |  |
| **2001** |  |  |  |  |
| **2002** |  |  |  |  |
| **2003** |  |  |  |  |
| **2004** |  |  |  |  |
| **2005** |  |  |  |  |
| **2006** |  |  |  |  |
| **2007** |  |  |  |  |
| **2008** |  |  |  |  |
| **2009** |  |  |  |  |
| **2010** |  |  |  |  |
| **2011** |  |  |  |  |
| **2012** |  |  |  |  |
| **2013** |  |  |  |  |

**Culture**

|  | **Did the laboratories use culture to confirm suspected cases? (Yes/No)** | **Number of cases confirmed by culture** | **% of cases confirmed by culture (among confirmed cases)** |
| --- | --- | --- | --- |
| **2000** |  |  |  |
| **2001** |  |  |  |
| **2002** |  |  |  |
| **2003** |  |  |  |
| **2004** |  |  |  |
| **2005** |  |  |  |
| **2006** |  |  |  |
| **2007** |  |  |  |
| **2008** |  |  |  |
| **2009** |  |  |  |
| **2010** |  |  |  |
| **2011** |  |  |  |
| **2012** |  |  |  |
| **2013** |  |  |  |

**Other**

|  | **Number of cases not confirmed by laboratories (clinical cases definition only)** |
| --- | --- |
| **2000** |  |
| **2001** |  |
| **2002** |  |
| **2003** |  |
| **2004** |  |
| **2005** |  |
| **2006** |  |
| **2007** |  |
| **2008** |  |
| **2009** |  |
| **2010** |  |
| **2011** |  |
| **2012** |  |
| **2013** |  |
